# Iatrogenic Anetoderma of Prematurity: A Case Report and Review of the Literature

**DOI:** 10.1155/2014/781493

**Published:** 2014-10-08

**Authors:** Laura Maffeis, Lorenza Pugni, Carlo Pietrasanta, Andrea Ronchi, Monica Fumagalli, Carlo Gelmetti, Fabio Mosca

**Affiliations:** ^1^NICU, Department of Clinical Sciences and Community Health, Fondazione IRCCS Ca' Granda Ospedale Maggiore Policlinico, University of Milan, Via della Commenda 12, 20122 Milan, Italy; ^2^Pediatric Dermatology Unit, Fondazione IRCCS Ca' Granda Ospedale Maggiore Policlinico, University of Milan, Via Pace 9, 20122 Milan, Italy

## Abstract

Anetoderma is a skin disorder characterized by focal loss of elastic tissue in the mid dermis, resulting in localized areas of macular depressions or pouchlike herniations of skin. An iatrogenic form of anetoderma has been rarely described in extremely premature infants and has been related to the placement of monitoring devices on the patient skin. Because of the increasing survival of extremely premature infants, it is easy to foresee that the prevalence of anetoderma of prematurity will increase in the next future. Although it is a benign lesion, it persists over time and can lead to significant aesthetic damage with need for surgical correction. Sometimes the diagnosis can be difficult, especially when the atrophic lesions become evident after discharge. Here, we report on a premature infant born at 24 weeks of gestation, who developed multiple anetodermic patches of skin on the trunk at the sites where electrocardiographic electrodes were previously applied. The knowledge of the disease can encourage a more careful management of the skin of extremely premature babies and aid the physicians to diagnose the disease when anetoderma patches are first encountered later in childhood.

## 1. Introduction

Anetoderma is a rare benign dermatosis characterized by focal loss of mid dermal elastic tissue, resulting in well-circumscribed areas of macular depressions or pouchlike herniations of skin [1–4]. The term* anetoderma* is derived from the Greek words* anetos *(relaxed) and* derma *(skin). Histological examination of the lesion typically reveals not only normal skin findings on hematoxylin-eosin staining, but also a significant reduction in elastic fibers within the dermis on Verhoeff-Van Gieson staining. The loss of elastic tissue could be caused by either decreased production or increased destruction of elastic fibers [5, 6].

Anetoderma may occur as a primary idiopathic phenomenon or secondary to many autoimmune, infectious, inflammatory, tumor, or drug-induced diseases (Table 1) [5, 6]. Historically, primary anetoderma was subclassified into two types, the Jadassohn-Pellizzari type and the Schweninger-Buzzi type, depending, respectively, on the presence or absence of prior inflammation at the site of the lesion. However, this classification is of historical interest because histologic features and prognosis are the same in both conditions [5, 6]. Among the primary forms of anetoderma, familial forms have been described [2, 7].

Anetoderma has rarely been reported in newborns. Both congenital and acquired iatrogenic forms have been described in preterm infants. The congenital form has been reported in babies born between 24 and 25 weeks of gestation and its origin is still unclear, even if a congenital defect in the production of elastic fibers in the dermis has been hypothesized [5, 6]. The acquired iatrogenic form has been reported in infants born between 24 and 32 weeks of gestation, who spent a long time in neonatal intensive care unit (NICU). Its origin has been related to the placement of monitoring devices (transcutaneous oxygen monitoring, electrocardiographic electrodes, temperature probes, adhesives, etc.) on the patient skin [3, 8–10]. Although it is a benign lesion, it persists over time and can lead to disfigurement with need for surgical correction.

Here, we report on a premature infant born at 24 weeks of gestation, who developed multiple anetodermic patches of skin on the trunk at the sites where electrocardiographic electrodes were previously applied.

## 2. Case Report

Our patient was born at 24 weeks of gestation by caesarean section. She was the second-born infant in a monochorionic diamniotic twin pregnancy. The twins' mother was well throughout the pregnancy. No history of infections in pregnancy was reported. Her twin sister died at one week of age because of a severe intraventricular haemorrhage. Her birth weight was 470 g, placing her between the 3rd and the 10th centile for weight. Her Apgar score was 3 at 1 minute. She was intubated at 1 minute and transferred to the NICU, where she was treated for respiratory distress and a patent ductus arteriosus which failed to close with medical treatment and required a surgical intervention. She developed a severe bronchopulmonary dysplasia requiring prolonged noninvasive ventilatory support.

**Figure 1:**
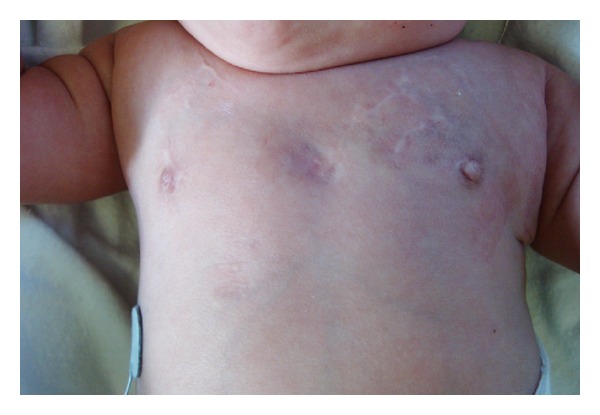
Ovalar, light-violaceous patches in the middle of upper chest. Translucent, coalescing, and bilateral lesions in subclavicular regions.

Numerous, localized, round-flat, and atrophic patches of skin on her upper chest were first noticed between the 4th and the 5th month of age while in NICU. All lesions were ventrally located and were between 5 and 15 mm in diameter. The largest lesions were localized in the middle of the chest and were ovalar, well-demarcated with a light-violaceous hue, without herniation (Figure 1). In the subclavicular areas, the lesions were less demarcated and more coalescing. Her skin was otherwise normal. None of the lesions was present at birth. Some bruise-like and ecchymotic lesions on the chest without necrosis or atrophy were described on medical records during the second week of extrauterine life. The location of the skin lesions corresponded to the sites of electrocardiographic electrodes placement.

The baby is now 7 months old and is still in NICU because of severe bronchopulmonary dysplasia, which is still requiring ventilatory support. Since their onset, the anetodermic lesions showed no changes and they are not yet evolved into the herniated anetoderma. Histological examination was not performed because of the very typical clinical presentation of the disease.

## 3. Discussion

Iatrogenic anetoderma of prematurity is clinically characterized by atrophic, round-flat or ovalar, skin-colored to violaceous, and brown or gray depressions or outpouchings of the skin, ranging from several millimeters to several centimeters in diameter. They are localized on the ventral surface of the chest, abdomen, upper arms, and proximal thighs, where monitoring leads or other medical devices are usually placed [3, 8–10]. Sometimes the atrophic lesions are preceded by erosive or ecchymotic patches, but in most cases previous skin lesions are absent or not diagnosed.

Iatrogenic anetoderma of prematurity was firstly described by Golden [9] in 1981. They reported on two premature infants, who developed anetoderma at transcutaneous oxygen monitoring sites. It was hypothesized that the intense heat under the probes could have caused a first-degree burn.

Subsequently, only 25 cases have been reported in the medical literature. Prizant et al. [3] reported on 9 cases of anetoderma in patients who were born between 24 and 29 weeks of gestation. The newborns developed anetodermic patches of skin on the trunk and the proximal extremities during their stay in NICU. The authors hypothesized that this acquired form of anetoderma could be due to the monitoring leads or adhesives tapes which were placed on the skin of the newborns.

Colditz et al. [8] described two infants born at 27 weeks of gestation who presented multiple lesions of anetoderma on the forehead at 3 months of age. The lesions appeared at the sides of gel electrocardiographic electrodes placement for electrical impedance tomography. Local hypoxemia due to pressure from the electrodes on immature skin was thought to be the cause of the disorder. The infants reported by Colditz were both growth-retarded (birth weight: 630 and 520 g). Reduced growth and thickness of the epidermis associated with intrauterine growth retardation may contribute to the formation of anetoderma.

This observation agrees with that of Todd [4] who reported an anetoderma associated with a monitoring lead in a severely growth-retarded twin (gestational age at birth: 32 weeks; birth weight: 794 g).

Ben-Amitai et al. [1] described two identical twins born at 26 weeks of gestation (birth weight: 1200 and 1050 g) who presented with a similar atrophic patch on the abdomen just lateral to the umbilicus at age 3 months. The authors suggested the possible role of genetic factors in the onset of the disease. However, since only two pairs of monozygotic twins concordant for anetoderma have been reported [1, 6], genetic factors probably are not pivotal.

The highest number of case series was reported by Goujon et al. [10] in 2010. Anetoderma was diagnosed clinically between the age of 6 weeks and 5 months in 11 preterm infants (gestational age at birth: 25–30 weeks; birth weight: 725–1250 g). Previous placement of monitoring leads was reported in most cases. Local hypoxemia due to pressure on immature skin or excessive traction on the skin when adhesive electrodes or tapes are removed was assumed to be the cause of the lesions. The thinness of the skin, the immaturity of its structure, an altered elastin metabolism, or an easier activation of elastolytic enzymes, such as metalloproteases, may give reason for the premature skin predisposition to anetoderma formation.

In our case, the correspondence between the site of involvement and placement of electrocardiographic leads was evident, since some lesions had the size and the shape of electrodes. Furthermore, the anetoderma was noticed when the patient was still in NICU and the electrocardiographic leads were still located on the lesional skin. No lesions were present at birth, so a congenital anetoderma was excluded. Our patient was growth-retarded and had an extremely low birth weight. Some authors believe that intrauterine and postnatal growth retardation may be more related to the onset of anetoderma than a low gestational age [4, 8]. The skin lesions were extensive and should be monitored over time to assess the severity of the aesthetic damage.

We believe that neonatologists, pediatricians, and dermatologists should be aware of iatrogenic anetoderma of prematurity. Firstly, the disease, although benign, persists over time and can lead to a significant aesthetic damage with psychological disorders of the patient and need for surgical correction. Secondly, the frequency of the disease, probably underestimated, is likely to increase given the increased survival of extremely premature infants.

The knowledge of iatrogenic anetoderma of prematurity can help neonatologists to prevent it, paying particular attention to the use of medical devices such as electrodes, adhesive tapes, and other medical stuff in order not to stress such an immature and predisposed skin, and can help pediatricians and dermatologists in a correct diagnosis when lesions become evident after discharge. In this case, a prolonged hospitalization in NICU and a thorough history regarding the presence or absence of skin lesions at birth may facilitate the differential diagnosis with congenital anetoderma and other dermatoses present at birth, such as Goltz syndrome (focal dermal hypoplasia), aplasia cutis congenita, and congenital erosive and vesicular dermatosis. Technological improvement has significantly increased neonatal survival, but it should match a higher “refinement of care,” that is, attention to detail, which can improve the quality of life.

## Figures and Tables

**Table 1a:** (a) Primary anetoderma (idiopathic)

Jadassohn-Pellizzari type (precedent clinical inflammation)	
Schweninger-Buzzi type (no precedent clinical inflammation)	
Familial	
Congenital	

**Table 1b:** (b) Secondary anetoderma (diseases associated with anetoderma)

(1) Autoimmune conditions	(i) Addison disease (ii) Antiphospholipid syndrome (iii) Discoid lupus (iv) Graves disease (v) Haemolytic anemia (vi) Sjögren syndrome (vii) Systemic lupus erythematosus (viii) Takayasu arteritis

(2) Infectious conditions	(i) Chicken pox (ii) HIV infection (iii) Leprosy (iv) Lyme disease (v) Molluscum contagiosum (vi) Syphilis (vii) Tuberculosis

(3) Inflammatory conditions	(i) Acne vulgaris (ii) Granuloma annulare (iii) Insect bites (iv) Mastocytosis (v) Prurigo nodularis

(4) Tumor/deposition conditions (benign and malignant)	(i) Cutaneous plasmacytoma (ii) Lymphocytoma cutis (iii) Melanocytic naevi (iv) Myxofibrosarcoma (v) Nodular amyloidosis (vi) Pilomatricomas (vii) Schwannomas (viii) Xanthomas

(5) Drug induced	(i) Penicillamine (ii) Hepatitis B vaccination

(6) Iatrogenic	Anetoderma of prematurity
